# Expression Profiling of EMT Transcriptional Regulators ZEB1 and ZEB2 in Different Histopathological Grades of Oral Squamous Cell Carcinoma Patients

**DOI:** 10.2174/0113892029284920240212091903

**Published:** 2024-02-15

**Authors:** Neha Baqai, Rafat Amin, Tehseen Fatima, Zeba Ahmed, Nousheen Faiz

**Affiliations:** 1 Dow Research Institute of Biotechnology and Biomedical Sciences, Dow University of Health Sciences, Ojha Campus, Karachi, Pakistan;; 2 Dow College of Biotechnology, Dow University of Health Sciences, Ojha Campus, Karachi, Pakistan;; 3 Otolaryngology, Dow Medical College-Dr.Ruth KM Pfau Civil Hospital Karachi, Dow University of Health Sciences, Karachi, Pakistan;; 4 Institute of Basic Medical Sciences, Dow University of Health Sciences, Ojha Campus, Karachi, Pakistan

**Keywords:** Epithelial-mesenchymal transition, moderately differentiated squamous cell carcinoma, oral squamous cell carcinoma, poorly differentiated squamous cell carcinoma, well differentiated squamous cell carcinoma, zinc finger E-box

## Abstract

**Background:**

Pakistan has a high burden of oral cancers, with a prevalence rate of around 9%. Oral Squamous Cell Carcinoma (OSCC) accounts for about 90% of oral cancer cases. Epithelial to Mesenchymal Transition (EMT) gets highly stimulated in tumor cells by adopting subsequent malignant features of highly invasive cancer populations. Zinc Finger E-Box binding factors, ZEB1 and ZEB2, are regulatory proteins that promote EMT by suppressing the adherent ability of cells transforming into highly motile cancerous cells. The present study aimed to analyze the expression of EMT regulators, ZEB1 and ZEB2, and their association with the clinicopathological features in different grades of OSCC patients.

**Methods:**

Tissue samples were collected for both case and control groups from the recruited study participants. Cancer tissues (cases) were collected from the confirmed OSCC patients, and healthy tissues (controls) were collected from third-molar dental extraction patients. The study participants were recruited with informed consent and brief demographic and clinical characteristics. The case group was further segregated with respect to the histological cancer grading system into well-differentiated (WD), moderately differentiated (MD), and poorly differentiated (PD) squamous cell carcinoma (SCC) groups. RNA was extracted from the tissue samples for expression profiling of ZEB1 and ZEB2 genes through quantitative real-time PCR (qRT-PCR).

**Results:**

All of the recruited participants had a mean age of 46.55 ± 11.7 (years), with most of them belonging to Urdu speaking ethnic group and were married. The BMI (kg/m^2^) of the healthy participants was in the normal range (18-22 kg/m^2^). However, BMI was found to be reduced with the proliferation in the pathological state of cancer. The oral hygiene of patients was better than the healthy participants, possibly due to the strict oral hygiene practice concerns of consultants. Every recruited OSCC patient had one or multiple addiction habits for more than a year. Patients reported health frailty (46.6%), unhealed mouth sores (40%), swallowing difficulties and white/reddish marks (80%), and restricted mouth opening (64.4%). Furthermore, 82.2% of the recruited patients observed symptoms within 1-12 months, and buccal mucosa was the most exposed tumor site among 55.6% of the patients. Expression profiling of EMT regulators showed gradual over-expressions of ZEB1 (8, 20, and 42 folds) and ZEB2 (4, 10, and 18 folds) in respective histological cancer grades.

**Conclusion:**

High expressions of ZEBs have been significantly associated with cancer progression and poor health. However, no association was found between OSCC with other clinicopathological features when compared to healthy controls.

## 1 INTRODUCTION

Oral cancer with an intemperate mortality rate is one of the abrasive types of human cancer [1]. GLOBOCAN 2020 stated that oral cancer cases have globally progressed with a 0.3 M incident rate and 0.1 M mortality rate per year [2]. Among them, the highest frequency is of lip and oral cavity cancers reported highly in south-central Asia, including Pakistan, India, and Sri Lanka [3]. The prevalence of oral cancer is approximately 9%, with an average range of 2 to 19% in the heterogeneous population of five big cities of Pakistan [4, 5]. The frequency of lip and oral cancer in the population of Karachi is approximately 30% [6]. Within two years (2017-2019), the Karachi Cancer Registry (KCR) has registered 33,309 cancer cases, among which 42.82% were male and 16.68% were female cancer patients of the lip and oral cavity [7]. Oral squamous cell carcinoma (OSCC), an epithelial malignancy of the oral cavity, accounts for above 90% of all oral cancer cases [8]. This pathological condition is influenced by various internal (developmental processes) and external (adaptive living) conditions [9-12]. OSCC arises from the epithelial lining of oral squamous cells invading nearby tissues of the mouth, tongue, or alveolar bone [13]. Regardless of advancements in available technologies, most of the OSCC patients are diagnosed at a later stage where the cancer has already been metastasized [14]. Even if the cancer has cured in earlier stages, the drug resistivity and re-occurrence can lead approximately one-third of the patients to advanced cancer stage [15]. The patient survival rate in such cancers nearly reduce to the average of five years [16].

Expression of specialized protein markers is a characteristic of cellular differentiation to the progenitor cell that follows cell discretion through Epithelial to Mesenchymal Transitions (EMT) [17]. EMT is a vital biological process of embryonic cell differentiation, in which epithelial cells gradually lose their morphology and identity by acquiring mesenchymal features [18]. Conversely, cells undergoing Mesenchymal to Epithelial Transition (MET) can reverse the acquired epithelial features [19-21]. However, tumorigenesis or cancer development is a pathological state in which a cell loses its identity by adapting tumor cell characteristics *via* bypassing apoptosis and hijacking the transcriptional and epigenetic controls [22-24].

EMT plays a substantial role in cancer progression by undergoing cell migration and metastasis, which is the reason for 90% death of cancer patients [25-27]. A number of signaling pathways, such as tyrosine kinase-associated pathways [28], TGFβ signaling [29], Notch signaling, Sonic Hedgehog (SHH) [30], WNT signaling pathways, and growth promotors, including fibroblast growth factor (FGF) [31], epidermal growth factor (EGF) [32], hepatocyte growth factor (HGF) [9], transforming growth factor b (TGFb) [33] and bone morphogenetic protein (BMP) [34] promoting EMT transitions are involved in this process.

Cell signaling pathways involved in the phenotypic switching of epithelial and mesenchymal transitions are under the regulatory control of various master regulators, including the TWIST family, SNAIL/SLUG family, ZEB1/ βEF1, ZEB2/ SIP1, and E12/E47 [35-37]. These transcriptional factors promote EMT by down-regulating the expression of the EMT regulator, *i.e*., E-Cadherin. The whole process disrupts the cellular adhesion property of the cell and causes it to remain detached and mobile in a mesenchymal state. Detached cells, through sequential transformation, adopt tumor characteristics and lead toward cancer progression. The role of these transcriptional factors has been studied in various cancers, including oral squamous cell carcinoma [38].

ZEB is an EMT transcriptional regulator belonging to the Zinc Finger E-box Binding Homeobox family [39]. It includes two closely related proteins: ZEB1 and ZEB2 [40]. The distinctive role of ZEB is to upsurge EMT towards sequential cancer progression and metastasis by dysregulating EMT transcriptional regulator E-Cadherin [41, 42]. Members of the ZEB family are not only involved in the cancer progression but also act as therapy resistance to the cancer that helps in escape and poor prognosis of the cancer [35].

In OSCC, higher expression of ZEB1 was reported to down-regulate the expression of E-cadherin, which causes loss of morphology of epithelial cells and has a crucial role in node metastasis in advanced oral cancer stages [43]. However, ZEB2, the second member of the ZEB family, is also specific to the E-box-like sequence and has the same function in EMT and tumor progression as ZEB1. It has been demonstrated that the high level of the ZEB2 mRNA is associated with poor patient prognosis and increased cancer stem cell-like properties [35].

Both ZEB1 and ZEB2 are homologous proteins. However, the transcription initiation site of ZEB1 lies near the start codon, while ZEB-2 has a transcription initiation site that lies more than 2.7 upstream of the start codon [22]. Overexpression of both ZEB1 and ZEB2 has been reported in a number of cancers, including prostate cancer [44, 45], breast cancer [46], gynecological cancers [47-49], head and neck cancers, and specifically in oral squamous cell carcinoma [29, 50, 51]. As there are no previous studies that revealed the expression of ZEB in the Pakistani population, the current study aims to determine the expression level of EMT transcription regulators (ZEB) in different grades of OSCC patients of the Pakistani population. The present study is based on molecular evaluation and thus determines the association between different etiological and pathological factors that may direct toward the screening of EMT markers ZEBs in the future for early diagnosis. The results of this study are expected to provide valuable insights into the OSCC treatment.

## 2 MATERIALS AND METHODS

### 2.1 Study Participants and Sample Recruitment

It is a case-control study with a ratio of 3:1 of OSCC cases to healthy controls with a total of 60 participants (n=60) with the approval of the Institutional Review Board (IRB) of Dow University of Health Sciences (DUHS), Karachi. The study subjects were recruited from 2020 to 2022 with informed signed consent, and their previous clinical history was obtained through a brief questionnaire. Healthy controls (n=15) were the patients appearing for third molar tooth extraction at the dental clinics of DUHS with no other clinical complications and cancer history. However, OSCC cases (n=45) were patients admitted for tissue biopsy and surgery at different health sectors of Karachi, including Indus Hospital, Karachi, and Dr. Ruth K. M. Pfau Civil Hospital Karachi. The patients were segregated on the basis of histological grading into well (n=15), moderately (n=15), and poorly differentiated (n=15) squamous cell carcinoma groups.

### 2.2 Human Tissue Sample Collection and Histopathology

Surgically excised tissue mass of ≤30 mg from recruited participants was collected into two separate micro-centrifuge tubes, one for RNA extraction containing RNA later and one for histopathology containing 10% formalin. Cancer tissue (cases) was excised from the OSCC patients, and noncancerous tissue (controls) was collected from third molar tooth extraction patients. Tissue samples were stored at -80°C for subsequent processing of RNA extraction.

### 2.3 RNA Extraction

RNA from the collected tissue samples of both cases and controls were extracted by an all-in-one DNA/RNA/protein Mini-preps kit (BIO BASIC, CAT#: BS88003) following the manufacturer’s protocol. Extracted RNA was then treated with an RNase-Free DNase kit (Promega) for the removal of any traces of DNA present following the manufacturer’s protocol. Furthermore, the optical density of each eluted sample was quantified at A260/280 using a micro-volume spectrophotometer, NanoDrop (Thermo Scientific 2000c). Both DNA and RNA samples were immediately stored at -20°C and -80°C, respectively.

### 2.4 Quantitative Real-time PCR (qPCR)

A total of 500 ng purified RNA was reversely transcribed for cDNA synthesis by Revert Aid First Strand cDNA synthesis kit (Thermo Scientific CAT#: K1622) by following the manufacturer’s protocol. Quantitative real-time PCR was performed in the QuantStudio-7 Flex Real-Time PCR Detection System, Applied Bioscience, with the specifically designed primers (Table **1**) for the targeted genes, ZEB1 and ZEB2. In contrast, GAPDH was used as the reference housekeeping gene for the endogenous control. Furthermore, qPCR amplification of each cDNA sample for the respective genes was performed in triplicate using 10µl reaction mixture by using cDNA, 2x PowerUP SYBER Green Master Mix and 10 µM of each forward and reverse primer for respective genes. Afterward, the final volume was made up of RNase-free water. The reaction mixture was kept for the hold stage at 50°C, 2 min and 95°C, 2 min, and for the PCR stage at 95°C, 1 sec and 60°C, 1 sec. Forty qPCR cycles were used for the amplification. The amplified expression of the targeted genes was determined by Relative Quantification (RQ) calculated through the proposed comparative ^2-∆∆^Ct method. MIQE guidelines were followed for the overall qPCR experimentation [52].

### 2.5 Statistical Analysis

Mean and standard deviation were reported for quantitative variables (normally distributed data) while median (IQR) was reported for qualitative variables (non-normally distributed data). Frequencies and percentages were calculated for all categorical variables. Pearson’s chi-square test was used for the comparison of baseline and clinicopathological characteristics of study participants. Kruskal-Wallis test and post-hoc by Dunnet’s test were used to compare the expression levels of ZEBs in different grades of OSCC patients, with a *p*-value of <0.05 considered significant.

## 3 RESULTS

### 3.1 Baseline Characteristics of the Study Participants

A total of 60 male participants were recruited in this case-control study. The cases (n=45) were categorized with international histological classification of tumor [51] into well, moderately, and poorly differentiated squamous cell carcinoma groups. However, healthy controls were individuals with no pathological conditions. Each study group consisted of 15 participants. The baseline data of the recruited participants are presented in Table **2**. Demographic data demonstrated that the mean age of study participants was 47 ± 11.70 (years). Age group categorization demonstrated that the highest 43.3% of participants were within 36-50 years of age group. The BMI (non-normal data) of the study participants was 18.13 (kg/m^2^) (IQR:16.3-34.4). BMI was also categorized according to WHO BMI cut-off ranges, which indicated that 43.3% of the individuals were in the normal BMI range, *i.e*., 18.5-24.9 (kg/m^2^). Among the study participants, 72% were married, and 53% belonged to Urdu speaking ethnic group.

Basic demographic characteristics of OSCC grades with a healthy control group were also compared by using Pearson's chi-square test. The statistical analysis showed no significant difference within age groups, marital status, and ethnicity, while body mass index (BMI) indicated a significant decrease with the upsurge of the cancer grade. The details of the comparison of baseline characteristics of different OSCC grades with healthy controls are briefly mentioned in Table **3**.

### 3.2 Clinicopathological Characteristics of OSCC Patients

Clinical features among recruited OSCC case groups were studied. It was found that 31.7% of the OSCC patients were habitual in consuming betel quid (*paan*). Among those, buccal mucosa was the most commonly affected site (55.6%) in OSCC patients and 46.6% of patients suffered from frailty (Table **4**).

### 3.3 Oral Health Status

The association of the clinical features within each case group (WDSSC, MDSCC, and PDSCC) was recorded with the confirmed tissue histopathology through H&E staining. The clinical representations of tumor sites are shown in Fig. (**1**). By brief clinical history, it was found that 27% of patients lost their teeth, 42% suffered from ear pain, 89% from mouth sores, and 80% with food swallowing difficulty and reported restricted mouth opening within the duration of 1-12 months. Among OSCC patients, the most common tumor site was buccal mucosa (56%). No association was reported by the chi-square test of the respective data sets of the case groups (Table **5**).

### 3.4 Expression Profiling of EMT Regulators *ZEB1* and *ZEB2*

The transcript levels of both *ZEB1* and *ZEB2* were over-expressed in patients’ groups as compared to the healthy controls. The mean fold change in *ZEB1* expression was found to be 8, 20, and 42 times elevated. However, *ZEB2* expression was 4, 10, and 18 times elevated in respective case groups of well, moderately, and poorly differentiated patients. Expression profiling of EMT regulators is shown in Figs. (**2** and **3**). A significant difference in terms of *ZEB1* and *ZEB2* expressions was found among OSCC groups. Furthermore, transcript levels of EMT regulators, ZEB1 and ZEB2, were further compared in OSCC groups with healthy controls, which also reported a significant difference in healthy control *vs*. well, moderate, and poorly differentiated groups, *i.e*., <0.001 (Table **6**).

### 3.5 Comparison of EMT Regulators *ZEB1* and *ZEB2* Expression

The study reported overexpression of *ZEB1* and *ZEB2* relative quantification in the median, *i.e*., 20.0 (IQR: 22.9-2.35) and 13.9 (IQR:15.4-1.5), respectively, as compared to healthy controls. The expression of both *ZEB1* and *ZEB2* was not associated with any of the patient’s characteristics, *i.e*., age, BMI, marital status, ethnicity, and oral habits (Table **7**).

## 4 DISCUSSION

OSCC is referred to as clinical and histopathological changes that are initiated from any minor injury till the development of the tumorous neoplasm through several precancerous phases [53]. The histopathological grading of the tumor is characterized based on the expansion of epithelial lesions during cancer development [54]. The epithelial changes include hyperkeratosis, hyperplasia, acanthosis or preneoplastic changes of dysplasia [55]. These changes vary in severity, being mild, moderate, and severe and play key roles in the development of cancer [56]. Oral cancer appears as epithelial dysplasia, followed by the abnormal proliferation of the squamous cells. This further causes the deterioration of the sub-epithelial basement (BM) and leads to local destruction and cancer invasion through metastasis. According to the two recent guidelines for tumor classification systems, namely patterns of tumor interstitial fluids [57] and international histological classification of tumor [58], the tumor lesion grading system is classified based on the histological grades as per the standard grading system into well, moderated, and undifferentiated or poorly differentiated tumor grades [59].

For the present study, the estimated sample size of each histological group was set to be n=15. To justify and validate study findings, only male OSCC patients were recruited to thoroughly examine the prevalence pattern of OSCC in male patients reported in most of the previous literature [59-62].

The present study confirmed the clinical and histopathological findings of the OSCC patients of different tumor grades, *i.e*., WDSCC, MDSCC, and PDSSC, compared to HC. Most of the patients were found to be in the age range between 36 to 50 years (Table **3**), which aligned with previously reported data on the Pakistani population, *i.e*., 46.7, S.D ± 10.2 years [59], 47.62, S.D ± 12.18 years [60], and 39.62 years [61]. The data ratifies that the onset of the disease occurs in the middle age individuals (36-50 years). An alternative reason could be late screening and diagnosis after the appearance of symptoms or due to inconsistent treatment patterns in OSCC patients. Nevertheless, contradictory to our results, some studies found deviations in the age ranges of OCSS patients. A comprehensive study from the Netherlands conducted from 1989 to 2018 reported that the incidence of OSCC has significantly increased by 2.5% (95% CI, 1.1-3.8) per year in younger patients of the age group 20-34 [59]. This might be due to early screening of the OSCC patients and/or differences in the effective social and geological environment.

The present study also evaluated Body Mass Index (BMI) in kg/m^2^ with respect to the histopathological grades of OCSS patients. It was found that 60% of poorly differentiated patients were underweight with <18.5 (kg/m^2^) BMI (Table **3**). Previous data demonstrated that the surgical excision of tumors in underweight patients needs critical care, and they suffer more from preoperative [63] and postoperative difficulties than other patients [64]. However, it was also reported that overweight OSCC patients have an increased risk of poor prognosis, re-occurrence, and patient mortality [65].

Some definite factors have been associated with the pathological state of OSCC, including oral hygiene, oral dependence habits, socioeconomic pressure, and ecological and communal factors [66]. Oral health patterns were also observed, and it was found that the recruited participants were habitual of consuming tobacco and had addictions to betel quid (*Paan*), *gutka*, areca nut, smoking, sweet suparis, *etc*., as mentioned in Table **4**. Due to these oral addictions, the buccal mucosa is the most prominent tumor site in such individuals [62, 65, 67, 68]. These findings were further confirmed in the present study, which are briefly illustrated in Fig. (**1**). Previous studies also identified that these oral habits, individually (*e.g*., areca nut alone) or in combination, are responsible for the initiation and progression of mouth cancers, *i.e*., OSCC [69, 70]. In OSCC patients, oral discomfort was another aspect of examining the disease condition. The present study reported certain oral distresses, including unhealed mouth sores, restricted mouth opening, and trouble in food swallowing (Table **5**). The current findings are in favor of previously reported literature [71-73], with pain being the prominent symptom in the suffering of OSCC patients.

**Fig. (2):**
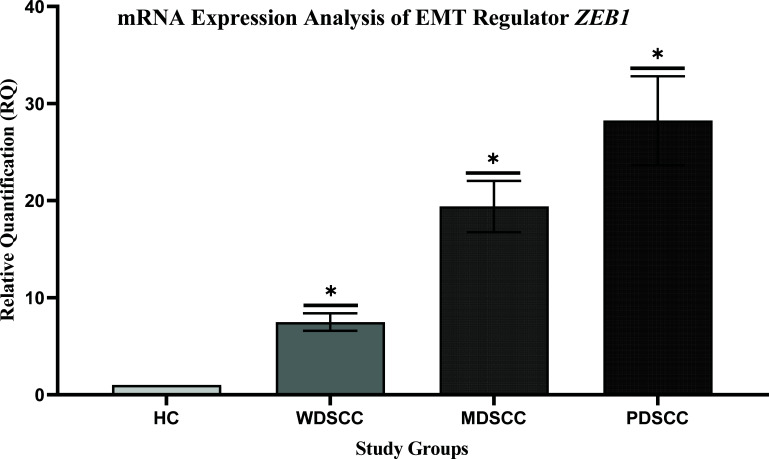
Expression profiling of EMT regulator ZEB1. Comparison of mRNA expression levels of ZEB1 in case groups of different tumor grades, well, moderately and poorly differentiated, with healthy controls. ***p-value < 0.001 was considered significant.

**Fig. (3):**
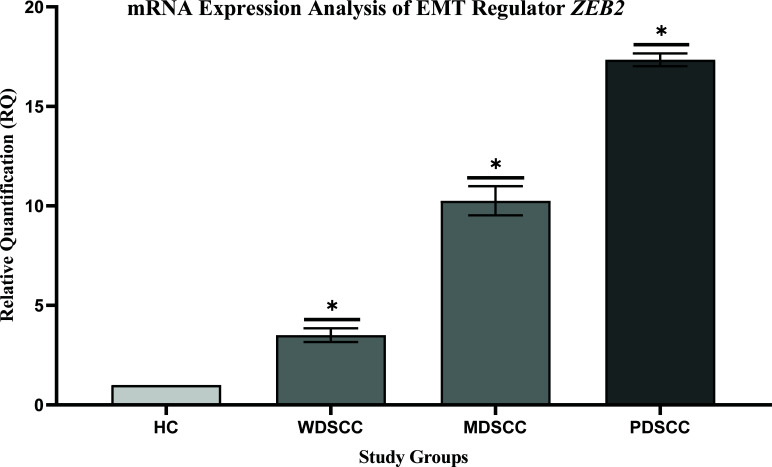
Expression profiling of EMT regulator ZEB2. Comparison of mRNA expression levels of *ZEB2* in case groups of different tumor grades: well, moderately and poorly differentiated, with healthy controls. ***p-value <0.001 was considered significant.

The stimulation of EMT is the primary mechanism by which the tumor cells acquire malignant features through suppressing E-cadherin protein and transform into a highly migratory and invasive cancer cell population [52]. There are abundant regulatory factors involved in the regulation of EMT. However, the present study has focused on only Zinc-finger E-box binding proteins, ZEB1 and ZEB2. The overexpression of ZEBs is known to alter normal functioning and suppress E-cadherin expression and is critically involved in cancer progression. This current study reported overexpression of both transcriptional regulators *ZEB1* and *ZEB2* (Figs. **2** and **3**). However, a significant difference was found in each tumor grade, WDSCC, MDSCC, and PDSCC, when compared to healthy controls. The expression profiling of these proteins was consistent with the reported literature, which detected over-expression of *ZEB1* [74-77] and *ZEB2* [78-80] in human cancers, including lung cancer, prostate cancer, colorectal cancer, and bladder cancer. Hence, the altered expressions of ZEBs could be used as prognostic signals in human cancers [81].

## CONCLUSION

The current study thoroughly examined the association between the clinicopathological features of OSCC patients of different grades with healthy control subjects. The data showed that among all the features, BMI has a significant effect on OSCC patients. It was found that as the tumor grade increased, the BMI decreased, indicating the poor health of the patients. Similarly, the expressions of ZEbs were gradually over-expressed with each cancer grade. However, no association was found with other clinical features of the study participants. This study provides evidence that as the OSCC progresses, the mRNA transcript levels significantly over-express from well-differentiated to poorly differentiated.

## Figures and Tables

**Fig. (1):**
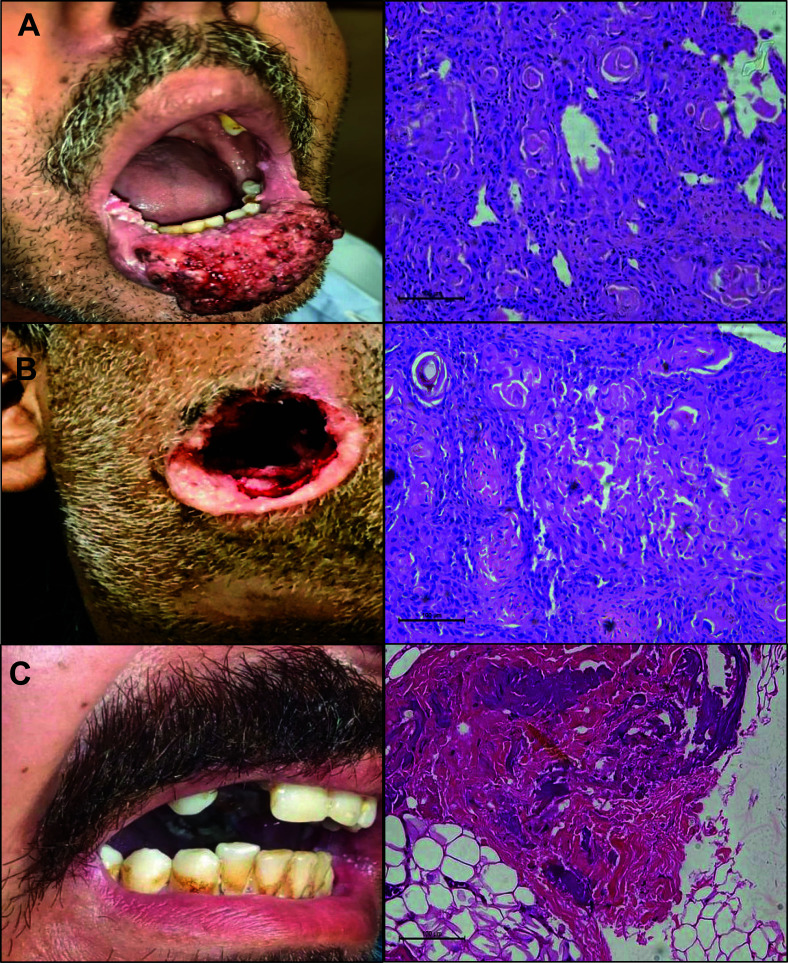
Clinical and histopathological representation of OSCC patients. Clinical and H&E stained slide representation of tumor grades: **A**) Well differentiated, **B**) Moderately differentiated, and **C**) Poorly differentiated squamous cell carcinoma case group recruited in the present study.

**Table 1:** Primers for real-time quantitative polymerase chain reaction (RT-PCR).

**Primer Name**	**Primer Sequence**	
**ZEB1**	Forward	**5-**’TGCACTGAGTGTGGAAAAGC-**3’**
	Reverse	***3’-*** *TGGTGATGCTGAAAGAGACG* ***-5’***
**ZEB2**	Forward	**5’-**CGCTTGACATCACTGAAGGA**-3’**
	Reverse	***3’-*** *CTTGCCACACTCTGTGCATT* ***-5’***
**GAPDH**	Forward	**5’-**AGGGCTGCTTTTAACTCTGGT**-3’**
	Reverse	***3’-*** *CCCCACTTGATTTTGGAGGGA* ***-5’***

**Table 2:** Baseline characteristics of the study participants (n=60).

**Characteristics**	**n (%)**
**Age Categories (years)**	**-**
20-35	15(25.0)
36-50	26(43.3)
>50	19(31.7)
**BMI Categories (kg/m2)**	**-**
<18.5	18(30.0)
18.5-24.9	26(43.3)
>25	16(26.7)
**Marital Status**	**-**
Single	17(28.3)
Married	43(71.7)
**Ethnicity**	**-**
Urdu	32(53.3)
Sindhi	12(20.0)
Punjabi	6(10.0)
Pathan	10(16.7)

**Table 3:** Comparison of OSCC grades with healthy controls according to the baseline characteristics (n=60).

**Characteristics**	**HC n=15 (%)**	**WDSSC n=15 (%)**	***p*-value (HI *vs* WD)**	**MDSCC n=15 (%)**	***p*-value (HI *vs* MD)**	**PDSCC n=15 (%)**	***p*-value (HI *vs* PD)**	**Total n=60 (%)**	**†Overall *p*-value**
**Age (years)**									
20-35	6(40)	3(20)	**0.295**	2(13.3)	**0.234**	4(26.7)	**0.514**	15(25)	**0.637**
36-50	4(26.7)	8(53.3)		7(46.7		7(46.7)		26(43.3)	
>50	5(33.3)	4(26.7)		6(40)		4(26.7)		19(31.7)	
**BMI (kg/m^2^)**									
<18.5	1(6.7)	4(26.7)	**0.113**	4(26.7)	**0.113**	9(60)	0.009*	18(30)	0.024*
18.5-24.9	6(40)	8(53.3)		8(53.3)		4(26.7)		26(43.3)	
>25	8(53.3)	3(20)		3(20)		2(13.3)		16(26.7)	
**Marital Status**									
Single	6(40)	2(13.3)	**0.090**	4(26.7)	**0.700**	5(33.3)	**0.999**	17(28.3)	**0.412**
Married	9(60)	13(86.7)		11(73.3)		10(66.7)		43(71.7)	
**Ethnicity**									
Urdu	10(66.7)	7(46.7)	**0.675**	7(46.7)	**0.317**	8(53.3)	**0.670**	32(53.3)	**0.704**
Sindhi	2(13.3)	4(26.7)		2(13.3)		4(26.7)		12(20)	
Punjabi	2(13.3)	2(13.3)		1(6.7)		1(6.7)		6(10)	
Pathan	1(6.7)	2(13.3)		5(33.3)		2(13.3)		10(16.7)	
Do not know	5(33.3)	4(26.7)		6(40)		2(13.3)		17(28.3)	

**Abbreviations:** HC: Healthy Controls; WDSCC: Well Differentiated Squamous Cell Carcinoma; MDSCC: Moderately Differentiated Squamous Cell Carcinoma; PDSCC: Poorly Differentiated Squamous Cell Carcinoma.

**Note:** *P*-value was calculated by using Pearson's Chi-square test and Fisher’s exact test.

**†**Overall *p*-value: Healthy Controls v/s Well, Moderately and Poorly Differentiated Squamous Cell Carcinoma.

*p-value <0.05 was considered as significant.

**Table 4:** Clinicopathological characteristics of OSCC patients (n=45).

**Characteristics**	**n (%)**
**Oral Habits (Yes)**	
Betal quid (*Paan*)	19(31.7)
Betal nuts (Areca nuts)	13(21.7)
Tobacco	6(10.0)
Gutka	21(35.0)
Sweets	3(5.0)
Supari	3(5.0)
Smoking	18(30.0)
Any other	12(20.0)
**Tumor Site**	
Lip and Tongue	14(13.1)
Buccal Mucosa	25(55.6)
Palate	6(13.3)
**Comorbidity (Yes)**	
Hypertension	6(10.0)
Diabetes	2(3.3)
Allergy	4(6.6)
Frailty	28(46.6)
Other conditions	2(3.3)

**Table 5:** Association of clinicopathological characteristics with OSCC grades (n=45).

**Characteristics**	**WDSCC n=15(%)**	**MDSCC n=15(%)**	**PDSCC n=15(%)**	***p*-value**
**Chewing Habits (Yes)**				
*Paan*	9(60.0)	4(26.7)	6(40.0)	0.177
Areca nuts	2(13.3)	2(20.0)	6(40.0)	0.209
Tobacco	2(13.3)	1(6.7)	3(20.0)	0.562
Gutka	8(53.3)	11(73.3)	2(13.3)	0.004**
Sweets	2(13.3)	1(6.7)	0(0.0)	0.343
Supari	2(13.3)	1(6.7)	0(0.0)	0.343
Smoking	8(53.3)	4(26.7)	5(33.3)	0.293
Any other	3(20.0)	4(26.7)	2(13.3)	0.659
**Clinical Condition (Yes)**				
Loose teeth	5(33.3)	6(40.0)	1(6.7)	0.092
Ear pain	8(53.3)	6(40.0)	5(33.3)	0.649
Sores that do not heal itself	13(86.7)	12(80.0)	15(100.0)	0.207
Difficulty in swallowing	11(73.3)	11(73.3)	14(93.3)	0.287
White/Reddish marks in the mouth	12(80.0)	12(80.0)	12(80.0)	0.091
Restricted mouth opening	9(60.0)	8(53.3)	12(80.0)	0.283
**Tumor Site**				
Lip and tongue	6(40.0)	5(33.3)	3(20.0)	0.677
Buccal mucosa	7(46.7)	9(60.0)	9(60.0)	
Palate	2(13.3)	1(6.7)	3(20.0)	

**Note:** *P*-value was calculated using Pearson's Chi-square test and Fisher’s exact test.

**p-value <0.05 was considered as significant.

**Table 6:** mRNA transcript levels of EMT regulators (ZEBs) in OSCC.

**MRNA Transcript Levels of ZEBs**	**ZEB1**		**ZEB2**	
	**Median IQR**	**†*p*-value**	**Median IQR**	**†*p*-value**
HC	0.0 (1.0-1.0)	<0.001***	0.0 (1.0-1.0)	<0.001***
WDSCC	1.5(8.0-6.4)		0.6(3.8-3.2)	
MDSCC	4.5(22.4-17.8)		1.3(10.7-9.4)	
PDSCC	8.8(31.9-23.1)		0.5(17.5-16.9)	
**Comparison of ZEBs’ mRNA Transcript Level *Vs* Healthy Control**	**-**			
WD	-	<0.001***	-	<0.001***
WDSSC		<0.001***		<0.001***
PDSSC		<0.001***		<0.001***

**Abbreviations:** HC: Healthy Controls; WDSCC: Well Differentiated Squamous Cell Carcinoma; MDSCC: Moderately Differentiated Squamous Cell Carcinoma; PDSCC: Poorly Differentiated Squamous Cell Carcinoma.

**Note:** *p*-value was calculated by using the Kruskal-Wallis test with post-hoc by Dunnet’s test.

**†**Overall *p*-value: Healthy Controls v/s Well, Moderately and Poorly Differentiated Squamous Cell Carcinoma

***p-value <0.001 was considered significant.

**Table 7:** Comparison of mRNA transcript levels of EMT regulators (ZEBs) with patients characteristics (n=45).

-	**ZEB1**		**ZEB2**	
	**Median (Q1-Q3)**	***p*-value**	**Median (Q1-Q3)**	***p*-value**
**Age (Years)**				
20-35	22.8(30.8-8.0)	**0.948**	13.9 (17.5-3.6)	**0.955**
36-50	16.7(24.7-8.0)		13.8 (17.1-3.3)	
>50	15.1(23.1-8.0)		13.1(16.9-3.8)	
**BMI (kg/m^2^)**				
<18.5	16.7(29.6-12.9)	**0.317**	10.8(17.4-6.6)	**0.432**
18.5-24.9	14.5(22.4-7.9)		6.9(10.7-3.8)	
>25	19.8(27.8-8.0)		12.5(15.8-3.3)	
**Marital Status**				
Single	11.8(29.6.-17.8)	**0.288**	8.0(17.4-9.4)	**0.232**
Married	15.1(23.1-8.0)		13.6(17.0-3.0)	
**Ethnicity**				
Urdu	16.8 (24.7-7.9)	**0.866**	13.4(17.1-3.7)	**0.835**
Sindhi	17.2(24.7-7.5)		13.4(17.1-3.7)	
Punjabi	18.7(26.7-8.0)		12.4(15.7-3.3)	
Pathan	13.1(26.0-12.9)		7.5(14.1-6.6)	
**Oral Habits (Yes)**				
*Paan*	15.2(23.1-7.9)	**0.355**	13.6(16.9-3.3)	**0.355**
Areca nuts	11.8(29.6-17.8)	**0.184**	8.0(17.4-9.4)	**0.144**
Tobacco	6.4(31-9-7.5)	**0.401**	13.8(17.5-3.7)	**0.314**
Gutka	14.5(22.4-7.9)	**0.137**	7.4(7.0-3.3)	**0.086**
Smoking	15.2(23.1-7.9)	**0.150**	13.3(16.9-3.3)	**0.190**
Any other	18.0(26.0-8.0)	**0.880**	10.5(14.1-3.6)	**0.880**

**Note:** *P*-value calculated by using the Kruskal-Wallis test; *p*-value <0.05 was considered significant.

## Data Availability

The authors declare that all the data supporting the findings of this study are available within the article.

## LIST OF ABBREVIATIONS

BMI: Body Mass Index
BMP: Bone Morphogenetic Protein
EGF: Epidermal Growth Factor
EMT: Epithelial to Mesenchymal Transition
FGF: Fibroblast Growth Factor
HGF: Hepatocyte Growth Factor
IRB: Institutional Review Board
KCR: Karachi Cancer Registry
MD: Moderately Differentiated
MET: Mesenchymal to Epithelial Transition
OSCC: Oral Squamous Cell Carcinoma
PD: Poorly Differentiated
qRT-PCR: Quantitative Real-time PCR
RQ: Relative Quantification
SCC: Squamous Cell Carcinoma
TGFb: Transforming Growth Factor b
WD: Well-differentiated
ZEB: Zinc Finger E-box

## STANDARDS OF REPORTING

STROBE guidelines were followed.
